# Doxorubicin-induced sinus node dysfunction associated with mitochondria and nuclear impairment in a mouse model

**DOI:** 10.1016/j.jphyss.2025.100047

**Published:** 2025-10-20

**Authors:** Kazuki Kobayashi, Mayu Nakatani, Yukihiro Harada, Yusuke Suzuki, Nahoko Fukunishi, Alphonse Boché, Tomoe Ueyama, Shu Nakao, Teruhisa Kawamura

**Affiliations:** a College of Life Sciences, Ritsumeikan University, 1-1-1 Nojihigashi, Kusatsu, Shiga 525-8577, Japan; b Research Institute for Material and Chemical Measurement, National Metrology Institute of Japan, National Institute of Advanced Industrial Science and Technology, 3-9-202 Tsukuba Central, 1-1-1 Umezono, Tsukuba, Ibaraki 305-8563, Japan; c Department of Life Science Support, Research Innovation Center, University Hospitals Sector, Tokai University, 143 Shimokasuya, Isehara, Kanagawa 259-1193, Japan; d Department of Physiology, Tokai University School of Medicine, 143 Shimokasuya, Isehara, Kanagawa 259-1193, Japan; e Research Organization of Science and Technology, Ritsumeikan University, 1-1-1 Nojihigashi, Kusatsu, Shiga 525-8577, Japan; f The Institute of Medical Sciences, Tokai University, 143 Shimokasuya, Isehara, Kanagawa 259-1193, Japan; g Ritsumeikan Global Innovation Research Organization, Ritsumeikan University, 1-1-1 Nojihigashi, Kusatsu, Shiga 525-8577, Japan

**Keywords:** Arrhythmias, Sinus node, Mitochondria, Animal model, Cardio-oncology

## Abstract

Doxorubicin (DXB), an effective anti-cancer drug, is well documented for its cardiotoxicity in the ventricular myocardium. Although DXB-induced cardiomyopathy can cause arrhythmias, its impact on the cardiac conduction system remains unclear. We aimed to investigate whether DXB affects the function and subcellular structure of the sinus node, the primary pacemaking site. C57BL/6 N mice received intraperitoneal injections of DXB at a total dose of 20 mg/kg. DXB treatment resulted in a reduced intrinsic heart rate during both the acute and chronic phases. We also observed DXB-induced downregulation of genes encoding pacemaker channels in both phases and Ca^2 +^ regulators in the chronic phase. Ultrastructural analysis revealed increased mitochondrial fragmentation, chromatin condensation, and a small number of severely damaged cells within the sinus node. These findings suggest that DXB impairs sinus node function, structure, and transcriptional regulation, potentially through mitochondrial and nuclear damage, leading to loss of pacemaker cells.

## Highlights

• Doxorubicin (DXB) reduces the intrinsic heart rate in mice.
• DXB induces downregulation of genes encoding heart rate regulators.
• DXB triggers mitochondrial and nuclear abnormalities in sinus node cells.
• These findings may guide targeted therapies to improve patients’ cardiac outcomes.

## Graphical Abstract

**Figure:**
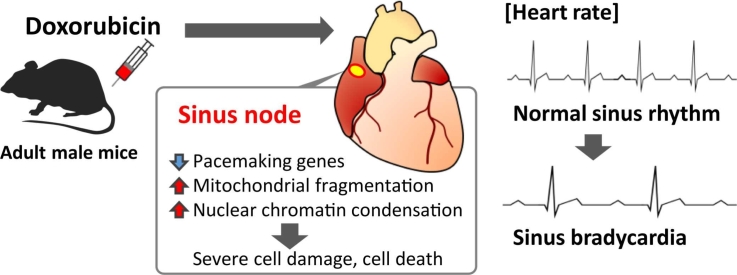

## Introduction

Doxorubicin (DXB) is a widely used and potent chemotherapeutic agent for the treatment of various malignant tumours. However, clinical use of DXB is limited by dose-dependent cardiotoxicity, which becomes particularly pronounced at the cumulative dosage of DXB exceeding 550 mg/m^2^ [1]. Currently, over 250 mg/m^2^ of DXB dosage is evaluated as a high risk of cardiovascular complications [2]. DXB-induced heart failure, also referred to as DXB-induced cardiomyopathy, is typically characterised by left ventricular dysfunction. This pathological condition often necessitates modification or discontinuation of anticancer therapy and requires additional cardiac care. Mechanistically, DXB cardiotoxicity has been attributed to DNA damage, excessive reactive oxygen species (ROS), mitochondrial dysfunction and topoisomerase II inhibition, ultimately leading to apoptosis in the ventricular myocardium [3].

While ventricular dysfunction is well documented in DXB-induced cardiomyopathy [2], the effect of DXB on the cardiac conduction system remains poorly understood. The conduction system is a specialised micro-tissue responsible for generating and propagating electrical impulses that highly coordinate pumping motion. The impulses are regularly generated in the sinus node (SN), the primary pacemaking region of the conduction system, and the electrical conduction continues to the ventricular myocardium that drives the heartbeat [4]. Various cardiac arrhythmias are attributed to functional and structural impairment of the conduction system. Previous studies have addressed that SN dysfunction causing bradycardia links to molecular remodelling of pacemaking ion channels resulting from congestive heart failure, endurance exercise, pulmonary hypertension, and diabetes [5]. Recent animal studies have reported that DXB induces electrophysiological impairments, including bradycardia, conduction disturbance, and increased susceptibility of atrial fibrillation [6], [7]; however, the effect of DXB on the conduction system remains poorly understood.

In the present study, we investigated whether DXB disrupts function and structure of the SN in a murine model of DXB-induced cardiomyopathy. Despite only mild myocardial remodelling, we found that DXB slowed intrinsic heart rate in both *in vivo* and *ex vivo* settings. Microscopy analyses of the SN revealed nuclear DXB accumulation, mitochondrial fragmentation, nuclear abnormalities, and a small population of damaged cells. Gene expression analyses demonstrated downregulation of genes responsible for pacemaking. These findings suggest that DXB contributes to pacemaker dysfunction through organellar damage and further transcriptional dysregulation, offering a new insight into the molecular basis of DXB-associated bradycardia.

## Materials and methods

### Ethic for animal study

All animal experiments in the present study were approved by the Institutional Animal Care and Use Committees at Ritsumeikan University and Tokai University. The procedures conformed to the Guidelines for Proper Conduct of Animal Experiments established by the Science Council of Japan, and the Fundamental Guidelines for Proper Conduct of Animal Experiment and Related Activities in Academic Research Institutions issued by the Ministry of Education, Culture, Sports, Science and Technology. Mice were housed in groups of two to five in a temperature- and humidity-controlled room (at 22–25 °C with 70 % humidity) with a 12-hour light/12-hour dark lighting cycle, with ad libitum access to food and water.

### Doxorubicin-induced cardiomyopathy modelling

After an acclimatisation period of more than one week in the animal facility, eight-week-old male C57BL/6 N mice were injected intraperitoneally with 15 mg/kg of DXB (Adriacin®, Sandoz, Tokyo, Japan) on day 0, and 5 mg/kg on day 14. Saline was administered to control mice as the vehicle. Body weight and general condition were monitored regularly throughout the experimental period. On day 21, after electrocardiogram recordings the mice were humanely euthanised by cervical dislocation and used for subsequent analyses.

### Histology and measurement of myocyte and fibrosis areas

The apex of the left ventricle and right atrial preparation containing the SN region were collected from freshly taken hearts. Tissue samples were embedded in OCT compound (Sakura Finetek, Tokyo, Japan) and rapidly frozen in cold isopentane. Frozen tissue blocks were sectioned to a thickness of 20 μm and were mounted onto silane-coated glass slides. To visualise the plasma membrane, the sections were fixed in 10 % neutral buffered formalin and incubated with Alexa Fluor 594-conjugated wheat germ agglutinin (Thermo Fisher Scientific, MA, USA) for 20 min. After washing with phosphate-buffered saline (PBS), the stained sections were coverslipped using VectaShield (Vector Laboratories, CA, USA). Images were acquired using a BZ-X710 inverted microscope equipped with a 20x objective (Keyence, Osaka, Japan). The cross-sectional are of ventricular myocytes was counted from randomly chosen 100–200 cells in the left ventricular free wall.

To identify the SN region, immunofluorescence labelling for hyperpolarisation-activated and cyclic nucleotide-gated channel 4 (HCN4), a predominant pacemaker channel, was performed on frozen sections of right atrial preparations. Briefly, sections were fixed in 10 % neutral buffered formalin in PBS and permeabilised in 0.1 % Triton-X-100 in PBS. After blocking with 0.1 % bovine serum albumin in PBS, the sections were incubated overnight at 4 °C with rabbit polyclonal anti-HCN4 antibodies (1:100, APC-052, Alomone Labs). Following PSB washes, the sections were incubated with Alexa Fluor 594-conjugated anti-rabbit IgG secondary antibodies (1:250, A-11012, Thermo Fisher Scientific) for 45 min at room temperature. Fluorescent micrographs were acquired using a confocal laser-scanning microscope (TCS SP5, Leica Microsystems) with LAS-AF software at excitation/emission wavelengths of 543 nm/570–620 nm.

For fibrosis assessment, sections were fixed in 10 % formalin/PBS, washed with PBS, and stained in Picrosirius red solution. After dehydration through a graded ethanol series, samples were coverslipped with Pathomount (Fujifilm Wako Pure Chemical, Osaka, Japan) and coverslip. Micrographs were obtained using a BZ-X710 inverted microscope equipped with a 20x objective. Fibrosis was quantified in five randomly chosen images of the left ventricular free wall.

### Electrocardiogram recording

To acclimatise mice to the custom-made electrode platform, electrocardiogram (ECG) recordings were performed on days 1, 4, 7, and 14. ECG signals were acquired using a PowerLab 26 T amplifier-integrate A/D converter and analysed with LabChart 8 software (ADInstruments, Dunedin, New Zealand). To determine intrinsic heart rate, ECG recordings were conducted on conscious mice under autonomic blockade on day 21, as previously described [8]. Autonomic blockade was achieved via intraperitoneal injection of 0.2 mg/kg propranolol hydrochloride, followed by 0.2 mg/kg atropine sulphate.

### *Ex vivo* extracellular potential recording

Immediately after euthanasia by cervical dislocation, the hearts were rapidly excised. To microdissect right atrial preparations containing the intact SN, the ventricles and the left atrial wall were trimmed away in an oxygenated Tyrode’s solution containing 100 mM NaCl, 4 mM KCl, 1.2 mM MgSO_2_, 1.2 mM KH_2_PO_4_, 1.2 mM CaCl_2_, 25 mM NaHCO_3_ and 11 mM D-glucose, bubbled with 95 % O_2_ and 5 % CO_2_ to give a pH of 7.4. For equilibration, right atrial preparations were placed in a perfusion bath that was continuously superfused with oxygenated Tyrode’s solution at 35–36 °C for 30 min. Extracellular potentials were then recorded for five minutes as the basal SN rate using a PowerLab 26 T system and LabChart 8 software (ADInstruments). To assess the effect of DXB on SN rate, the perfusion solution was replaced with Tyrode’s solution containing 30 μM DXB, and recording was continued for a further 40 min. Subsequently, the effect of 1 μM ivabradine (Tokyo Chemical Industry, Tokyo, Japan) on SN rate was measured for an additional 20 min following either vehicle or DXB treatment. The effect of 20 μM hydrogen peroxide on SN rate was also examined following equilibration. SN rate was determined by averaging the cycle length over a period of continuous 30–60 s.

### Tissue culture

A right atrial preparation was dissected in a pH-adjusted warm Tyrode’s solution containing 140 mM NaCl, 5.4 mM KCl, 0.45 mM MgCl_2_, 0.33 mM NaH_2_PO_4_, 5 mM HEPES, 5 mM D-glucose, and 1.8 mM CaCl_2_, with a pH of 7.4. Immediately after dissection, the preparation was transferred and pinned to a silicon-based dish containing tissue culture medium. The medium comprised Advanced DMEM/F-12 (Thermo Fisher Scientific) supplemented with 10 % foetal bovine serum (Cosmo Bio, Tokyo, Japan), 2.0 mM L-glutamine, and 5x penicillin–streptomycin (Nacalai Tesque, Kyoto, Japan), as previously described [9]. Preparations were then incubated at 37 °C in 5 % CO_2_ on an orbital shaker at 60 rpm. After a 3-hour equilibration period, the culture medium was replaced with medium containing 10 μM DXB. Saline was used as the vehicle control. Some preparations were subsequently used for cell isolation, as described below.

### Isolation and imaging of SN cells and atrial myocytes

After 20 h of tissue culture, SN cells and atrial myocytes were isolated from the right atrial preparations using an enzymatic digestion protocol. The digestion solution contained 1.0 mg/mL protease type XIV (Merck, Darmstadt, Germany), 0.1 mg/mL trypsin (Merck), 11.0 mg/mL collagenase type II (Worthington Biochemical, NJ, USA), and 0.3 mg/mL elastase (Worthington Biochemical) dissolved in a high-potassium and calcium-free Kraft Brühe (KB) buffer, as previously described with minor modification [10]. Following digestion, the preparations were washed with KB buffer and employed dissociation by gentle pipetting. The isolated cells in KB buffer were kept on ice until imaging. These cells were then transferred to a glass-based dish and imaged using a confocal laser-scanning microscope (FV3000, Evident, Tokyo, Japan). DXB fluorescence was detected at excitation/emission wavelengths of 543 nm/570–620 nm. Images were acquired at a resolution of 512 × 512 pixels using FV31S-SW Viewer software (Evident).

### Quantitative PCR

Tissue samples from the SN region and the right atrial appendage, taken from fresh hearts, were homogenised in TRIzol reagent (Thermo Fisher Scientific, MA, USA) and stored at −80 °C until further processing. Total RNA was extracted using a standard phenol-chloroform method. RNA yields were determined using a Nanodrop 2000 spectrophotometer (Thermo Fisher Scientific), and 250 ng of total RNA was reverse transcribed using ReverTra Ace (Toyobo, Osaka, Japan). Quantitative PCR was then performed on a StepOnePlus Real-Time PCR System (Thermo Fisher Scientific), and mRNA expression levels were normalised using 18S expression levels.

### Transmission electron microscopy

Tissue strips (approximately 1 ×3×0.2 mm) of the right atrial wall containing the SN region were dissected from fresh hearts under a stereo microscope. Tissue strips from the right atrial appendage were also microdissected to assess differences between the SN and the atrial myocardium. Immediately after dissection, samples were processed for transmission electron microscopy, as previously described [11]. In brief, tissue pieces were fixed in 2 % paraformaldehyde and 2 % glutaraldehyde in 0.1 M phosphate buffer (pH 7.2) for 48 h, followed by post-fixation in 1 % osmium tetroxide-containing 0.05 M phosphate buffer at 4 °C for 2 h. The samples were then dehydrated through a graded ethanol series, substituted with 100 % acetone, and embedded in epoxy resin (Quetol 812, Nissin EM). Semi-thin sections were stained with 1 % toluidine blue and observed under a light microscope to reveal the SN region or longitudinally aligned atrial myocytes. Ultrathin sections were then sliced and stained with uranyl acetate and lead citrate. Ultrastructural analysis was performed using a JEM-1400 transmission electron microscope (JEOL, Tokyo, Japan), operating at an accelerating voltage of 80 kV.

### Mitochondria phenomics analysis

The analysis of mitochondrial morphology relies on the individual segmentation of mitochondria from transmission electron microscopy images. Based on our previous work on cellular segmentation [12], mitochondria morphology was assessed using seven to nine images per condition, each containing 50–150 mitochondria, resulting in a total of 567–1431 mitochondria per condition segmented using *napari-cellpose* [13]. Segmentation was facilitated using the *empanada* plugin in *napari* [14], and a deep learning model developed in our previous work [12] was employed to improve segmentation accuracy. Manual correction of the label shapes was performed afterward to ensure high-quality segmentation.

Mitochondrial shapes were subsequently processed using the open-source software *FIJI* [15] with a custom macro specifically designed to extract individual mitochondrial shapes [12]. Principal component analysis was then conducted using *Celltool*[16] to acquire a ‘mean’ contour by merging all mitochondrial shapes. Each individual contour was compared to this mean, determining values for size relative to the overall mitochondrial population. Finally, for each of the four conditions, kernel density estimation was used to visualise for comparison of the distributions of mitochondrial size across groups.

### Statistical analysis

After confirming the variances, unpaired or paired *t*-tests was used for the comparison between two groups, and one-way ANOVA followed by Games-Howell nonparametric post-hoc test was used for the comparison between four groups. Statistical significance was calculated using R packages including Stats and Rosetta or Microsoft Excel. All the data are expressed as mean ± SD, and *p* < 0.05 was considered significant.

## Results

### Doxorubicin-induced cardiomyopathy modelling in mice

**Fig. 1:**
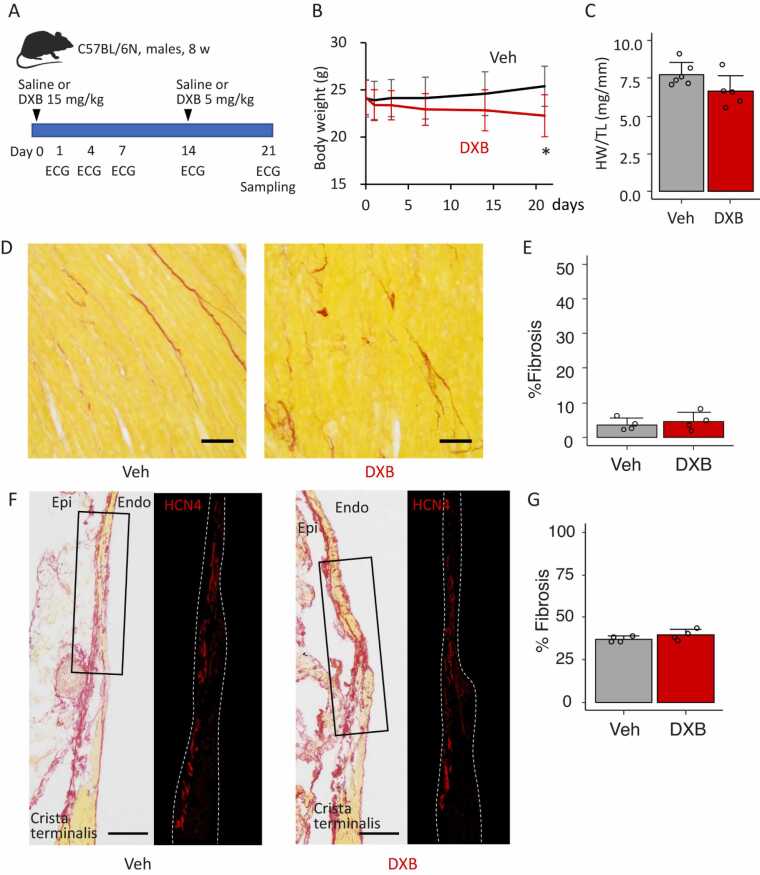
Doxorubicin-induced cardiomyopathy modelling in mice. A. A work flow of the modelling of doxorubicin (DXB)-induce cardiomyopathy in C57BL/6 N adult male mice. Saline was injected as a vehicle (Veh) control. B. The time course of the body weight in Veh- and DXB-treated mice. While the body weight of Veh mice incrementally gained, that of DXB mice decreased. n = 6 in Veh and 10 in DXB. C. Heart weight-to-tibial length ratio in Veh- and DXB-treated mice. n = 6 in Veh and 5 in DXB. D and E. Picrosirius red-stained sections (D) and averaged fibrosis area (E) of the left ventricular myocardium in Veh- and DXB-treated mice. n = 4/group. Scale bars = 20 μm. F. Representative picrosirius red-stained sections of the SN region in Veh- or DXB-treated mice and enlarged area labelled with HCN4, a pacemaker channel as a SN marker, indicated by rectangles in picrosirius red images. Scale bars = 100 μm. **G.** Averaged fibrosis area of the SN region in Veh- and DXB-treated mice. n = 4/group. * *p* < 0.05 vs Veh, determined by unpaired *t*-test.

To produce the DXB-induced cardiomyopathy model, we intraperitoneally injected DXB to adult male mice at 15 mg/kg as the initial dosage on day 0 and an additional 5 mg/kg on day 14 (Fig. 1A). Saline was injected to control mice. The body weight in DXB-treated mice exhibited a gradual decrease, whereas vehicle-treated mice incrementally gained weight (Fig. 1B; n = 6 in vehicle and 9 in DXB, unpaired *t*-test, *p* < 0.05 vs vehicle on day 21). After euthanised with cervical dislocation, the heart weight was measured, and we found that the heart was slightly lighter in DXB-treated mice compared to control (Fig. 1C; n = 6 in vehicle and 5 in DXB). Interstitial fibrosis in the ventricular myocardium was quantified in histology, and fibrotic areas were similar in vehicle- and DXB-treated mice (Fig. 1D and E, n = 4 mice/group). We next assessed fibrosis in the SN region, identified by HCN4 immunolabelling, a predominant pacemaker channel. The SN region is intrinsically more fibrotic than the ventricular myocardium; however, DXB treatment did not induce additional fibrosis in this region (Fig. 1F and G). Ventricular myocyte size was also analysed in cross sections stained with red fluorescence-conjugated wheat germ agglutinin labelling the plasma membrane and exhibited no difference between the groups (Figs. S1A and S1B, n = 6 in vehicle and 5 in DXB). To examine pathological remodelling in the ventricular myocardium, we examined gene expression levels of the following sarcomere proteins: alpha-cardiac actin encoded by *Actc1* as a general myocardium marker, alpha-myosin heavy chain encoded by *Myh6* as an adult-type sarcomere marker, and beta-myosin heavy chain encoded by *Myh7* as a foetal- and pathological-type sarcomere marker. Quantitative PCR revealed that similar expression levels of *Actc1* and *Myh6* were observed in vehicle and DXB groups, whereas pathological *Myh7* was slightly upregulated by DXB treatment (n = 6 in vehicle and 5 in DXB; unpaired *t*-test, *p* = 0.072; Fig. S1C). These results, therefore, suggest that DXB treatments caused modest gene expression changes reflecting the slight myocardial remodelling.

### DXB slowed intrinsic resting heart rate in mice

**Fig. 2:**
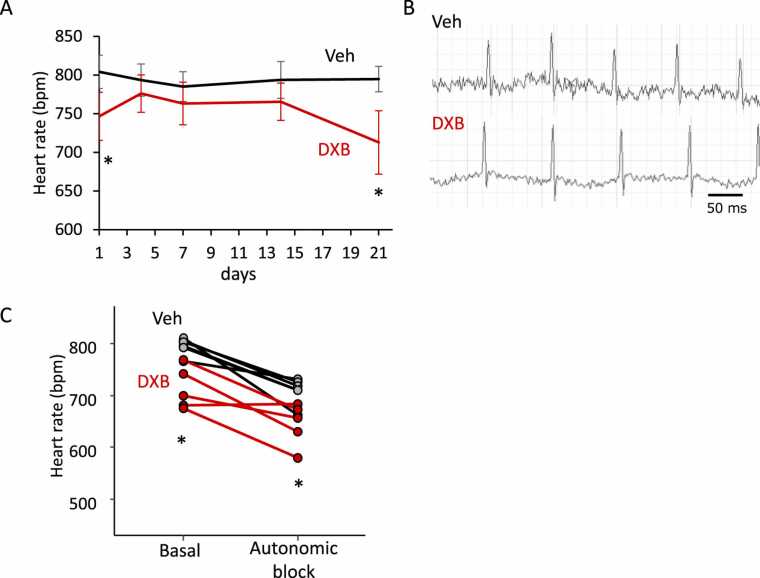
Doxorubicin slowed the resting heart rate *in vivo*. A. Heart rate changes during the doxorubicin (DXB)-induced cardiomyopathy modelling. Vehicle (Veh) is a saline injection control. B. Representative electrocardiogram recordings in Veh- and DXB-treated conscious mice after autonomic blockade with atropine and propranolol injections. C. Heart rate values before and after autonomic blockade, under which the intrinsic heart rate was determined, in Veh- and DXB-treated mice. n = 6 in Veh and 5 in DXB, respectively. *p < 0.05 vs Veh determined by unpaired *t*-test.

To address the effect of DXB on heart rate in conscious mice, ECG recordings were performed in mice treated with vehicle or DXB. Heart rate in laboratory mice we used for conscious ECG are usually acclimatised after three recordings, so that heart rate in those mice normally exhibits constancy from the fourth attempt and onwards. In the present study, the first three-time recordings on day 1, day 3, and day 7 in control mice (n = 6) revealed a gradual decrease in heart rate, while ECG recordings on day 14 and day 21 showed the heart rate constant. Compared to the control mice, DXB mice (n = 5) obviously presented a lower heart rate throughout the experimental period (unpaired *t*-test, *p* < 0.05 vs vehicle on days 1 and 21). In particular, on day 21, heart rate in conscious mice was obviously lower in the DXB than in the vehicle groups (Fig. 2A). We next investigated the effect of DXB on the intrinsic heart rate in conscious mice. To determine the intrinsic heart rate, autonomic blockade was employed with injections of propranolol and atropine. We observed significantly lower heart rate in DXB-treated animals (n = 5) compared to control animals (n = 6, unpaired *t*-test, *p* < 0.05) (Fig. 2B and C), suggesting that DXB induces SN dysfunction in mice.

**Fig. 3:**
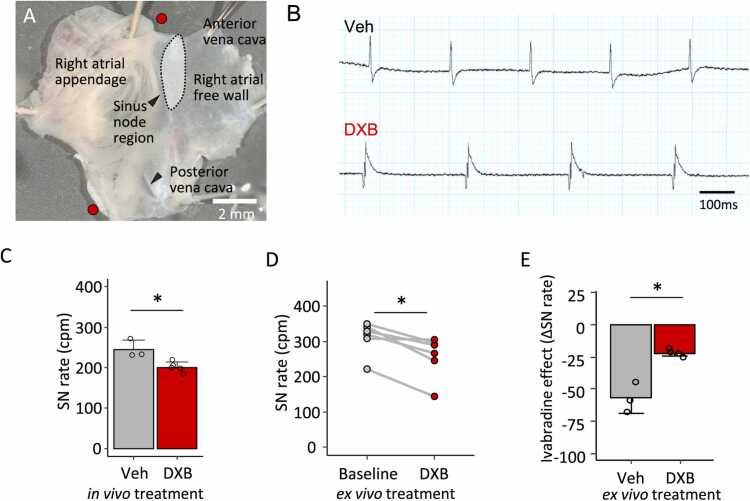
Doxorubicin (DXB) slowed the resting heart rate *ex vivo*. A. A micrograph of a right atrial (RA) preparation containing the sinus node. Red circles indicate electrode positions for an extracellular potential recording. B. Representative traces of the extracellular potential in RA preparations from vehicle (Veh)- and aDXB-treated mice. C. Mean sinus node spontaneous firing rate (SN rate) in Veh- and DXB-treated mice. n = 3 in Veh and 5 in DXB. D. SN rate changes in RA preparations before and after superfusion with a DXB-containing Tyrode’s solution. n = 5. **E.** Ivabradine, an HCN blocker, effect on SN rate in RA preparations after treatment with Veh or DXB *ex vivo*. n = 3 in Veh and 4 in DXB. *p < 0.05 determined by unpaired (C and E) and paired (D) *t*-tests.

We further analysed whether DXB treatment reduces the spontaneous firing rate in the isolated SN tissue. We prepared right atrial preparations, which were quickly dissected from a fresh mouse heart and superfused in a physiological Tyrode’s solution oxygenised with 95 % O_2_/5 % CO_2_ at 35–36 °C (Fig. 3A). As right atrial preparations are completely denervated and not exposed to circulating molecules that influence heart rate, they are well-suited for measuring the intrinsic heart rate reflected on cardiac automaticity [8]. The spontaneous SN rate recorded from right atrial preparations becomes stable after a 30-minute equilibration period; however, it is typically lower than the heart rate recorded *in vivo* by electrocardiogram due to a lack of sympathetic stimuli and circulating factors that increase heart rate. In control animals (n = 3), SN rate was 245.0 ± 22.8 counts per minute (cpm), whereas it was significantly lower in preparations from DXB-treated mice (Figs. 3B and 3C; n = 5, 201.0 ± 12.7 cpm, unpaired *t*-test, *p* < 0.05 vs vehicle). We also tested an acute effect of DXB on the SN rate *ex vivo*. DXB-containing Tyrode’s solution was superfused after equilibration in a perfusion chamber. The SN rate was significantly decreased by DXB treatment (Fig. 3D; n = 5, paired *t*-test, *p* < 0.05). These results appear to represent the heart rate changes observed *in vivo*; that heart rate was decreased at the acute (on day 1) and chronic (on day 21) phases, respectively (Fig. 2A and C). In addition, we examined the effect of ivabradine, a selective HCN channel inhibitor, on SN rate following vehicle or DXB treatment. The rate reduction induced by ivabradine was attenuated in DXB-treated preparations (Fig. 3; n = 3 in vehicle and 4 in DXB, unpaired *t*-test, *p* < 0.05 vs vehicle), suggesting that pacemaker function had already been inhibited by DXB. We also tested whether oxidative stress, as a potential cardiotoxic mechanisms of DXB, could influence intrinsic heart rate. Treatment of right atrial preparations with 20 μM hydrogen peroxide significantly reduced the SN rate (Fig. S2; n = 4, paired *t*-test, *p* < 0.05), suggesting that spontaneous firing is sensitive to excessive oxidative stress.

**Fig. 4:**
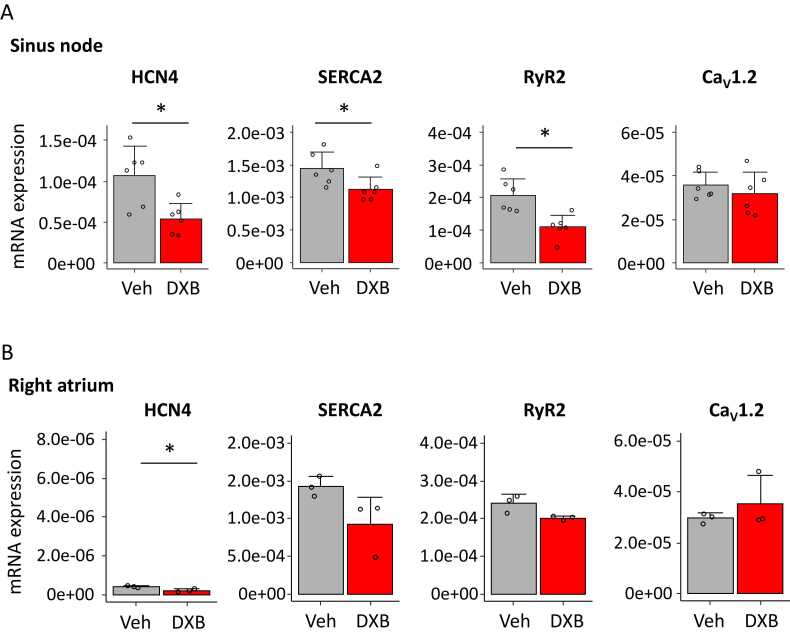
Chronic effect of doxorubicin (DXB) on expression levels of genes responsible for pacemaking. A and B. Gene expression levels of HCN4 pacemaker channel and Ca^2+^ regulators in the sinus node (A) and the right atrial myocardium (B) in vehicle (Veh)- and DXB-treated mice. n = 6 in SN, n = 3 in right atrium, *p < 0.05 vs Veh determined by unpaired *t*-test.

To address a molecular aspect underlying the heart rate reduction by DXB, we analysed expression levels of genes related to spontaneous firing of action potential in SN (n = 6) and atrial (n = 3) samples from DXB-treated mice. We found significant reduction of expression levels of HCN4, a pacemaker channel, in DXB samples. As highly expressed calcium regulators, the sarcoplasmic/endoplasmic reticulum calcium pump (SERCA2) and RyR2, an intracellular calcium release channel, were also downregulated by DXB treatment (unpaired *t*-test, *p* < 0.05 vs vehicle), whereas a voltage-gated calcium channel (Ca_V_1.2) gene was not affected (Fig. 4A). In the atrial samples, DXB tended to modestly decrease the expression levels of SERCA2 and RyR2, and it did not change the expression levels of Ca_V_1.2. Although HCN4 were downregulated (unpaired *t*-test, *p* < 0.05 vs vehicle), its expression levels were substantially at a low level compared to in the SN (Fig. 4B). We further tested the gene expression levels at the acute phase. In samples taken at 24 h after an intraperitoneal injection of vehicle or DXB at 15 mg/kg (n = 8/group), SN exhibited significant reduction of HCN channel gene. The other genes showed a tendency of DXB-induced downregulation, but these changes did not reach statistically significant (Fig. S3A). DXB did not affect transcript levels in AM samples (Fig. S3B). Although transcription levels of these ion transporters represent only an indirect aspect of protein function regulating heart rate, we speculate that transcriptional impairment of pacemaker channels and calcium regulators might be involved in DXB-induced bradycardia.

### DXB impaired nuclear component and mitochondria dynamics

**Fig. 5:**
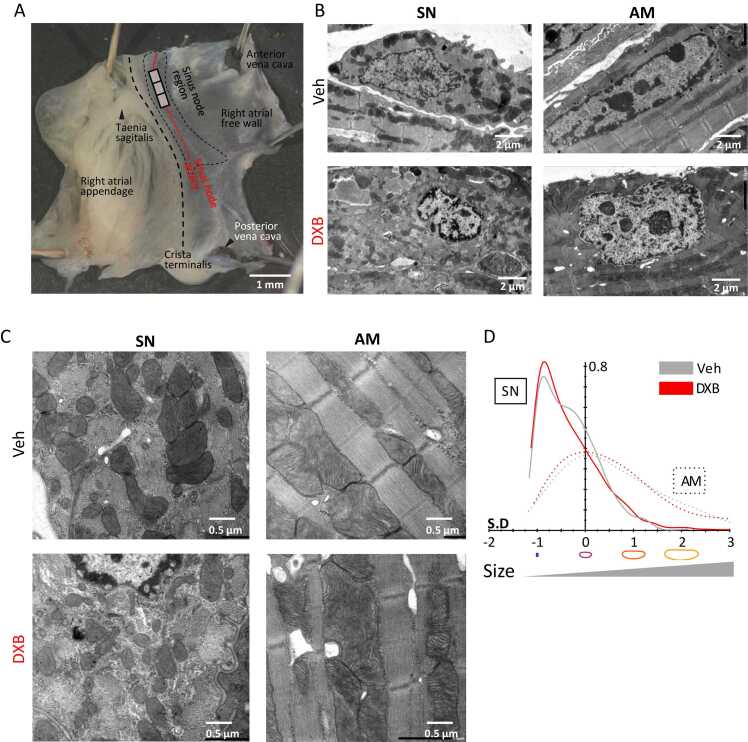
Doxorubicin (DXB) impaired nucleus and mitochondria in sinus node cells (SN) and atrial myocytes (AM). A. A micrograph of a right atrial preparation containing the SN region. The labels indicate anatomical landmarks for the SN region. Rectangles indicate the SN region for transmission electron microscopy. B and C. Representative electronmicrographs of SN and AM with *ex vivo* treatment of vehicle (Veh) or DXB (n = 3/group). In DXB group, coarse patterns of nuclear chromatin in SN and AM (B) and fragmented mitochondria in SN cells (C) are observed, respectively. D. Kernel density estimation of mitochondrial size distributions in SN and AM with or without DXB treatment.

DXB-induced cardiomyopathy presents as systolic cardiac dysfunction due to mitochondrial impairment and excessive oxidative stress followed by apoptosis in the ventricular myocardium [3]. To address the effect of DXB on the subcellular structure in the SN, transmission electron microscopy was employed in DXB- or vehicle-treated right atrial preparations (n = 3/group). To precisely collect the SN region, we used the crista terminalis, the taenia sagittalis, the anterior vena cava, and the sinus node artery as anatomical landmarks (Fig. 5A). Electron micrographs revealed that DXB affected nuclei and mitochondria in SN cells. Specifically, in DXB-treated preparations, DXB treatment increased condensation of the euchromatin in the nuclei and subnuclear membrane was observed throughout SN and atrial myocytes (Fig. 5B). To determine intracellular accumulation of DXB, we isolated SN cells and neighbouring atrial myocytes from right atrial preparations, which were pre-treated with vehicle or DXB in a tissue culture condition. As DXB contains red fluorescence, DXB accumulation is detectable under fluorescence observation. In SN and atrial myocytes, DXB obviously stained the nuclear membrane and euchromatin and slightly stained a few small particles in the cytoplasm, whereas other subcellular structures, including mitochondria, were not detected (Fig. S4; n = 3/ group). Furthermore, we observed that mitochondria fragmentation was evident in SN cells in DXB-treated preparations compared to control s (Fig. 5C). Quantitative analysis based on mitochondrial segmentation indicated that DXB treatment modestly shifted the size distribution of mitochondria in SN cells towards smaller populations (Fig. 5D). In atrial myocytes, the distribution was comparatively less affected (n = 567–1431 mitochondria from 3 animals per group; One-way ANOVA followed by Games-Howell post hoc test, p < 0.01 in SN and p < 0.05 in atrial myocytes for vehicle vs. DXB, respectively). We further found that a few numbers of SN cells were severely damaged by DXB, manifested by raptured myofibrils and mitochondrial swelling (Figs. S5A and S5B). These results suggest that DXB affects the structure of the nucleus and mitochondria in the SN. This may cause cell death and impaired energy supply, leading to pacemaker dysfunction.

In addition, to examine the structural consequence in this DXB-induced bradycardia model, transmission electron microscopy was employed in SN and atrial samples from vehicle- or DXB-treated mice on day 21 of the protocol. We observed DXB-treated SN with a coarse chromatin pattern, but other components including mitochondria and myofibrils and were similar between vehicle and DXB samples. Notably, a small number of cells were apparently damaged containing larger mitochondria with a lower cristae density, indicating mitochondrial swelling, and dilated sarcoplasmic reticulum. Atrial myocytes exhibited no considerable finding caused by DXB administration (Figs. S6A through S6C; n = 570–1065 mitochondria from 3 animals per group; One-way ANOVA followed by Games-Howell post hoc test, all conditions were *p* < 0.001 vs SN-vehicle), suggesting that these structural features reflect DXB-induced alterations of SN function and transcription.

## Discussion

In the present study, we investigated functional and structural alterations in the SN—the primary cardiac pacemaker—in a mouse model of DXB-induced cardiomyopathy, aiming to elucidate molecular mechanisms underlying DXB-associated bradycardia. Despite only mild myocardial remodelling, DXB-treated mice exhibited a reduced intrinsic heart rate in both *in vivo* and *ex vivo* settings. Ultrastructural analysis revealed mitochondrial fragmentation and nuclear abnormalities in SN cells following acute DXB exposure, and a small population of damaged cells during both the acute and chronic phases. In parallel, expression levels of key pacemaking genes appeared to be affected by DXB in the SN. Collectively, these findings suggest that DXB impairs SN function through combined structural damage and transcriptional dysregulation, ultimately leading to pacemaker dysfunction and bradycardia.

DXB is documented to disrupt intracellular ultrastructure in ventricular myocytes, including myofibrillar loss, cytoplasmic vacuolization, and sarcoplasmic reticulum dilation [17], [18], [19]. In a control condition, our electron microscopy analysis revealed that control SN cells exhibited irregularly arrayed myofibrils and mitochondria with relatively sparse cristae, in contrast to atrial myocytes, which contained highly organised structures. Although most DXB-treated SN cells did not display the overt structural damage comparable to that reported in ventricular myocytes, we observed increased mitochondrial fragmentation, coarse chromatin patterns, and several damaged cells in the SN.

Mitochondrial fragmentation is known to occur early in the apoptotic cascade and results from fission driven by apoptotic proteins such as B-cell lymphoma 2 (Bcl-2)-associated X-protein (BAX) [20]. Under normal conditions, BAX is inhibited by anti-apoptotic factors such as Bcl-2; however, under pathological stress—such as oxidative damage that downregulates Bcl-2—BAX becomes activated and promotes mitochondrial outer membrane permeabilisation [21], [22]. BAX also facilitates dynamin-related protein 1 (Drp1)-mediated fragmentation, further progressing apoptotic processes [20], [23]. Thus, the mitochondrial fragmentation observed in DXB-treated SN cells may reflect activation of intrinsic apoptotic pathways.

Additionally, our ultrastructural analysis displayed chromatin condensation in the subnuclear membrane in DXB-treated SN cells, where DXB accumulation was detected (Fig. S4). DXB is known to induce genomic instability and compromise nuclear membrane integrity via excessive ROS production [3], [24]. Such disruption could facilitate aberrant nucleocytoplasmic transport, potentially enhancing the translocation of pro-apoptotic factors such as cytochrome c. The abnormal chromatin organisation observed in DXB-treated SN cells further supports the idea that nuclear dysfunction contributes to the apoptotic signalling. Collectively, these results suggest that DXB may induces bradycardia, at least in part, through cell death triggered by mitochondrial fragmentation and nuclear damage in SN cells.

Excessive ROS production is a hallmark of DXB-induced cardiotoxicity, causing both cellular and molecular stress [25]. In ventricular myocytes, excessive ROS inhibits topoisomerase II, an essential enzyme responsible for breaking and resealing DNA strands during replication to regulate chromatin dynamics [26]. Inhibition of topoisomerase II impairs DNA repair that leads to nuclear and mitochondrial DNA damage, subsequently promoting apoptosis and metabolic dysfunction. DXB is also known to trigger the opening of the membrane permeability transition pore, leading to depolarisation of the mitochondrial membrane potential, Ca^2+^ overload, and activation of apoptotic pathways. Additional evidence suggests that DXB induces mitochondrial iron accumulation via dysregulation of iron transporters such as mitoferrin-2 and ABCB8, resulting in ROS overproduction via the Fenton reaction [27]. While these mechanisms have been characterised primarily in ventricular tissue, the present study revealed mitochondrial disruption in several SN cells, suggesting that similar ROS-related pathways may contribute to SN dysfunction. Our tissue electrophysiological experiment, showing that hydrogen peroxide markedly reduced spontaneous firing, further supports the role of ROS in this impairment. Although direct evidence of ROS involvement in SN cells in the context of DXB treatment remains limited, studies from other models support this possibility. In a murine heart failure model, chronic angiotensin II treatment led to sinus bradycardia associated with excessive ROS accumulation in the SN [28]. This oxidative stress activated calmodulin-dependent kinase II, promoting dysregulated intracellular calcium signalling, fibrosis, and cell death in the SN. Given that our imaging analysis revealed nuclear accumulation of DXB in SN cells, it is plausible that DXB increases ROS production and contributes to ROS-mediated cell death within this pacemaker tissue. As mitochondria are the primary sites of oxidative phosphorylation and energy production, excessive ROS is tightly linked to mitochondrial dysfunction. Future studies quantifying intracellular ROS and ATP levels, as well as assessing mitochondrial function in SN cells following DXB treatment, will be crucial to clarify the contribution of oxidative stress to DXB-induced SN dysfunction.

Examining the time course of resting heart rate following DXB administration in mice, we observed a slowing of heart rate during both the acute and chronic phases (Fig. 2A). At these stages, gene downregulation was detected in HCN4 pacemaker channel at both phases and in several Ca^2+^ regulators during the chronic phase, respectively (Figs. 4 and S3). Mechanistically, these heart rate changes likely reflect impaired function of the coupled-clock system comprising the membrane and Ca^2+^ clocks [29]. The HCN channel, which generates the pacemaker current underlying slow diastolic depolarisation, is a key component of the membrane clock. Reduced pacemaker current would be expected to prolong diastolic depolarisation and delay Ca^2+^ channel-mediated depolarisation, thereby decreasing the spontaneous firing rate. This interpretation is supported by our *ex vivo* experiment using a selective HCN channel inhibitor (Fig. 3E). During the chronic phase, downregulation of Ca^2+^ regulators such as SERCA2 and RyR2 could impair the Ca^2+^ clock, by reducing Ca^2+^ uptake and release from the sarcoplasmic reticulum—events that trigger pacemaker action potentials. Further investigation into the effects of DXB on ion channel function and intracellular Ca^2+^ regulation—such as analyses of pacemaker current density and Ca²⁺ dynamics—as well as on transcriptional regulation will be essential to address the molecular mechanisms underlying DXB-induced bradycardia.

From a pharmacokinetic view of blood DXB concentration, a previous study demonstrates that intraperitoneal injections of DXB at 12 mg/kg resulted in the peak concentration 2 h after administration, declined to approximately 40 % at 24 h, with similar kinetics across multiple organs [30]. More recently, an *in vivo* tracking study using an implantable biosensor demonstrated that intravenous injection of DXB at 10 mg/kg reaches a peak concentration at 2 h and returns to baseline levels by 48 h [31]. Based on these data, the acute DXB effect on heart rate observed in our study likely occurred when a substantial amount of DXB remained in circulation and cardiac tissues, whereas the chronic bradycardic effect appeared after DXB had been eliminated from the body. These observations suggest that the acute effect may involve direct interaction of DXB with pacemaking proteins or their transcriptional regulators, whereas the chronic effect may arise from structural damage or loss of pacemaker cells.

Various protocols for DXB-induced cardiomyopathy have been previously established, differing in dose and duration to mimic either acute or chronic cardiotoxicity. An acute model employing a single intraperitoneal injection of 25 mg/kg DXB was used to investigate molecular effects of DXB within 16–72 h, revealing DNA damage in ventricular myocytes [32]. In contrast, chronic models have involved weekly injections of 5 mg/kg over five weeks, leading to pronounced left ventricular dysfunction [27]. In our study, we employed a modified protocol consisting of 15 mg/kg followed by 5 mg/kg two weeks later, based on preliminary findings that higher repeated doses resulted in excessive mortality. Although our protocol is milder and shorter than typical chronic models, it effectively reduced intrinsic heart rate—our primary aim—while avoiding severe ventricular remodelling. Thus, despite its relatively moderate impact on ventricular function, our model successfully captures SN dysfunction without inducing severe cardiac failure.

In recent years, the interdisciplinary field of cardio-oncology has emerged to address the cardiovascular complications associated with cancer therapy. Accumulating evidence, including comprehensive guidelines, has become increasingly available for both health professionals and scientists in this field [2]. While numerous anticancer drugs have been developed, their cardiotoxicity has also been identified [33]. Compared to DXB-induced cardiomyopathy, the incidence of bradycardia—possibly resulting from SN dysfunction—appears particularly more common with microtubule inhibitors (e.g., paclitaxel), antimetabolites (e.g., 5-fluorouracil, cytarabine), alkylating agents (e.g., cisplatin) [34], [35], [36], anaplastic lymphoma kinase (ALK) inhibitors (e.g., crizotinib, alectinib, brigatinib, ceritinib), immunomodulatory drugs (e.g., thalidomide, pomalidomide) [2], [37], [38], and mitogen-activated protein kinase (MAPK) inhibitors such as inhibitors against rapidly accelerated fibrosarcoma (RAF) (e.g., dabrafenib, encorafenib) and MAPK kinase (MEK) (e.g., trametinib) [39], [40], [41]. Despite these clinical observations, the mechanisms underlying chemotherapy-induced SN dysfunction remain poorly defined. Further studies characterising the molecular and structural features of the conduction system in cancer patients treated with these drugs are essential for advancing both diagnostic precision and cardioprotective strategies. Moreover, as the present study included only male mice, it is important to note that clinical studies have reported sex-specific risks of anthracycline-induced cardiotoxicity [42]. For example, prepubertal female cancer survivors treated with anthracyclines have been shown to be at increased risk [43].Modelling chemotherapy-induced arrhythmias in both male and female animals will therefore be essential for elucidating sex-dependent susceptibilities.

In conclusion, our multidisciplinary investigation demonstrates that DXB-induced impairment of mitochondrial dynamics and nuclear architecture appears to promote DNA damage and activate cell death pathways as well as transcriptional dysregulation, ultimately contributing to SN dysfunction. This underscores the need for further research into the effects of anticancer drugs on the cardiac conduction system. Taken together, these findings highlight the emerging importance of pacemaker tissue investigation within the expanding field of cardio-oncology.

## Author contributions

SN and TK designed the study. KK, MN, YS, NF, TU and SN conducted the experiments. KK, NM, YH, AB, TU, SN and TK analysed the data. KK, AB, SN and TK prepared the figures and drafted the manuscript. KK, YH, TU, SN, and TK critically revised the manuscript for important intellectual content. All the authors approved the final version of the manuscript, and agree to be accountable for all aspects of the work in ensuring that questions related to the accuracy or integrity of any part of the work are appropriately investigated and resolved; and all persons designated as authors qualify for authorship, and all those who qualify for authorship are listed.

## CRediT authorship contribution statement

**Kazuki Kobayashi:** Writing – review & editing, Writing – original draft, Visualization, Methodology, Investigation, Formal analysis, Data curation. **Teruhisa Kawamura:** Writing – review & editing, Writing – original draft, Visualization, Validation, Supervision, Resources, Project administration, Methodology, Funding acquisition, Formal analysis, Conceptualization. **Shu Nakao:** Writing – review & editing, Writing – original draft, Visualization, Validation, Supervision, Project administration, Methodology, Investigation, Funding acquisition, Formal analysis, Data curation, Conceptualization. **Tomoe Ueyama:** Writing – review & editing, Methodology, Investigation, Formal analysis, Data curation. **Alphonse Boché:** Methodology, Formal analysis, Data curation. **Nahoko Fukunishi:** Methodology, Formal analysis, Data curation. **Yusuke Suzuki:** Methodology, Formal analysis, Data curation. **Yukihiro Harada:** Writing – review & editing, Validation, Formal analysis. **Mayu Nakatani:** Methodology, Investigation, Formal analysis, Data curation.

## Consent for publication

Not applicable.

## Ethics approval and consent to participate

This work was conducted under Institutional Guidelines of Care and Use of Laboratory Animal in Ritsumeikan University and Tokai University. Institutional Ethics Committee Approval number was #241098.

## Funding

This work was supported by JSPS KAKENHI Grants including 24K10018 (SN) and 24K22041 (TK); Mochida Memorial Foundation (SN); 2024 Tokai University School of Medicine Research Aid (SN); and Tokai University Research Organization Grant for Start-up 2023–11 (SN).

## Declaration of Competing Interest

The authors declare the following financial interests/personal relationships which may be considered as potential competing interests. Shu Nakao reports financial support was provided by Japan Society for the Promotion of Science. Teruhisa Kawamura reports financial support was provided by Japan Society for the Promotion of Science. Shu Nakao reports financial support was provided by Mochida Memorial Foundation for Medical and Pharmaceutical Research. If there are other authors, they declare that they have no known competing financial interests or personal relationships that could have appeared to influence the work reported in this paper.

## Data Availability

The data in this study is available from the corresponding authors upon reasonable request.

## Glossary

ALK: anaplastic lymphoma kinase
BAX: Bcl-2-associated X-protein
Bcl-2: B-cell lymphoma 2
DXB: doxorubicin
ECG: electrocardiogram
HCN: hyperpolarisation-activated and cyclic nucleotide-gated channel
KB: Kraft Brühe
MAPK: mitogen-activated protein kinase
MEK: mitogen-activated protein kinase kinase
PBS: phosphate buffered saline
RA: right atrium
RAF: rapidly accelerated fibrosarcoma
ROS: reactive oxygen species
SN: sinus node
Veh: vehicle

## Footnotes

Appendix A Supplementary data associated with this article can be found in the online version at doi:10.1016/j.jphyss.2025.100047.
