# Supplementary material for: Doxorubicin-induced sinus node dysfunction associated with mitochondria and nuclear impairment in a mouse model
Source: J Physiol Sci. 2025 Oct 20;75(3):100047. doi: 10.1016/j.jphyss.2025.100047 (PMC12596622; doi:10.1016/j.jphyss.2025.100047)
Supplement: Supplementary file 1 — Supplementary material [file mmc1.pdf]

Supplementary materials

Doxorubicin-induced sinus node dysfunction associated with mitochondria and nuclear impairment in a mouse model

Kazuki Kobayashi, Mayu Nakatani, Yukihiro Harada, Yusuke Suzuki, Nahoko Fukunishi, Alphonse Boché, Tomoe Ueyama, Shu Nakao, Teruhisa Kawamura

Fig. S1

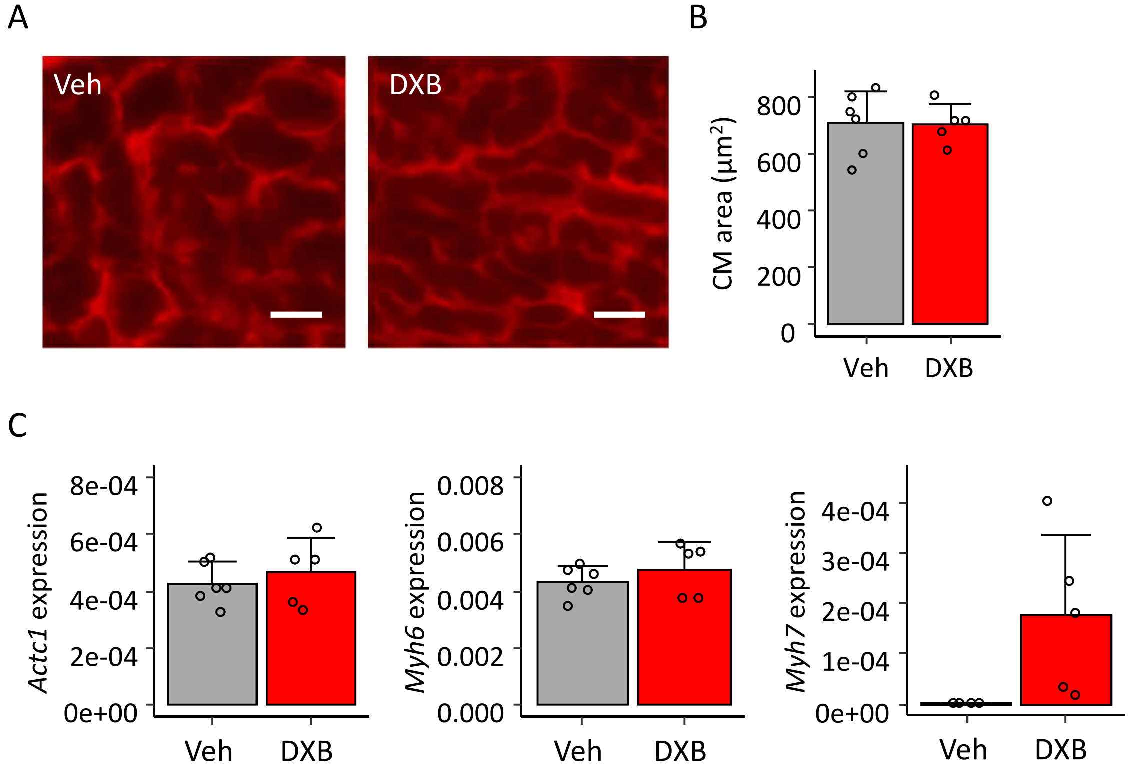

Fig. S1. Doxorubicin (DXB) causes mild myocardial remodelling in mice.
A and B. Fluorescent micrographs of cross-sections labelled with Alexa Fluor 594-conjugated wheat germ agglutinin (A) and averaged cross-sectional area (B) of cardiomyocytes in the left ventricular myocardium in vehicle (Veh)- and DXB-treated mice. n = 6 in Veh and 5 in DXB. Scale bars = 20 µm. **C.** Transcript levels of cardiac contractile proteins including alpha cardiac actin (*Actc1*), α-myosin heavy chain (α-MHC, *Myh6*) and β-MHC (*Myh7*) in left ventricular myocardium from Veh- and DXB-treated mice. n = 6 in Veh and 5 in DXB, *p* values were determined by unpaired *t*-test.

Fig. S2

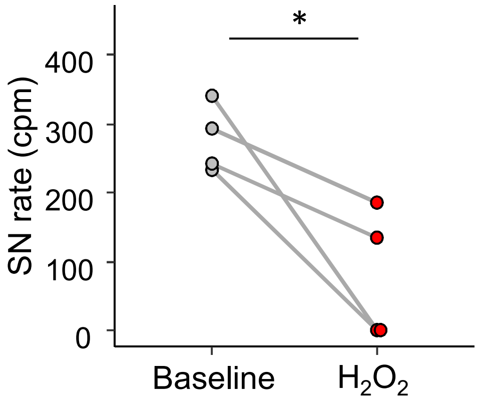

Fig. S2. Spontaneous firing in the sinus node (SN) is sensitive to excessive oxidative stress.
SN rate changes in RA preparations before and after superfusion with a 20 μM hydrogen peroxide (H_2O_2)-containing Tyrode's solution. n = 4. *$p < 0.05$ determined by paired *t*-test.

Fig. S3

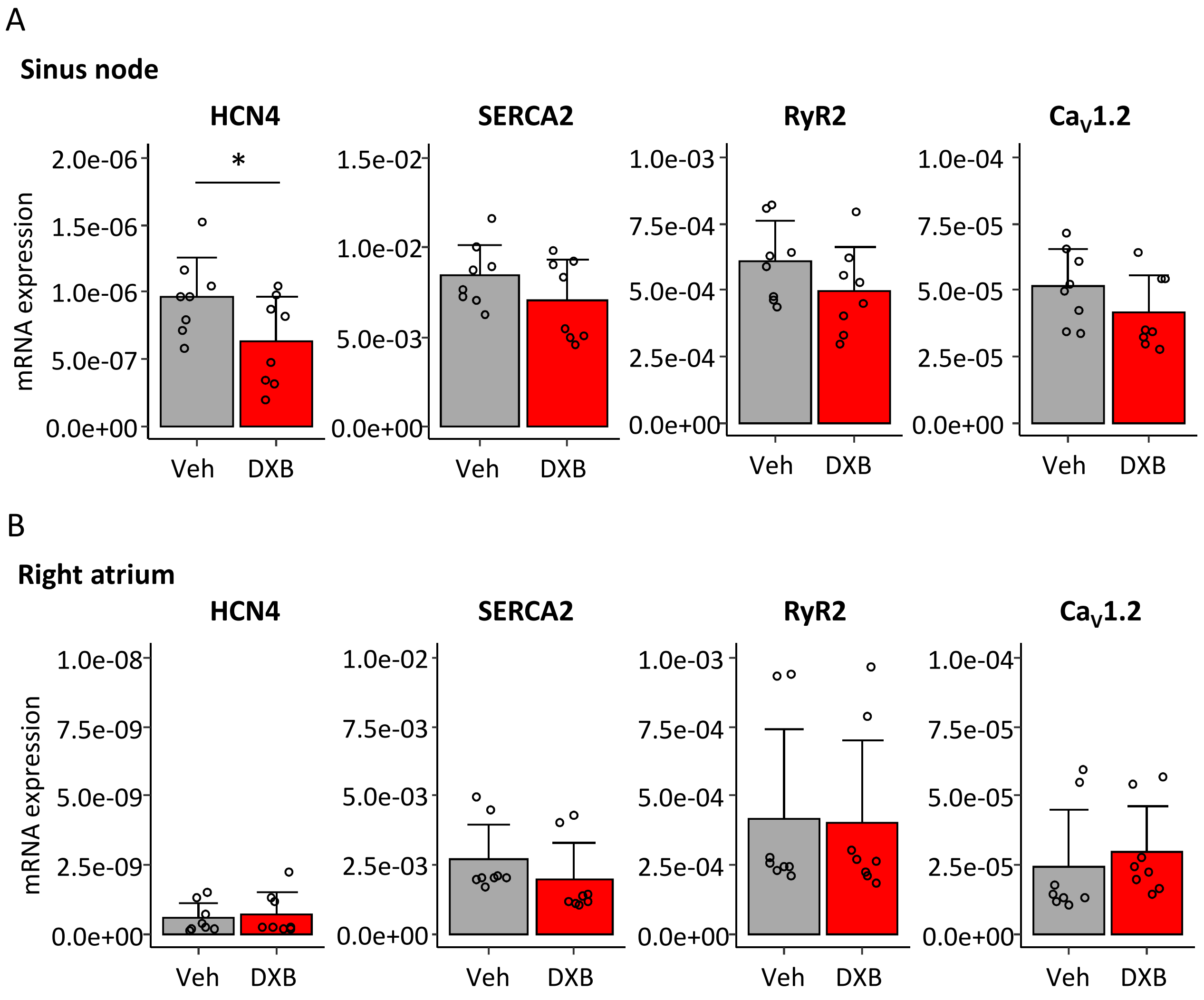

Fig. S3. The acute effect of doxorubicin (DXB) on expression levels of genes responsible for pacemaking. A and B. Gene expression levels of HCN4 pacemaker channel and Ca^{2+} regulators in the sinus node (A) and the right atrial myocardium (B) in mice 24 hours after treatment with vehicle (Veh) or DXB. n = 8/group, *$p < 0.05$ vs Veh determined by unpaired *t*-test.

Fig. S4

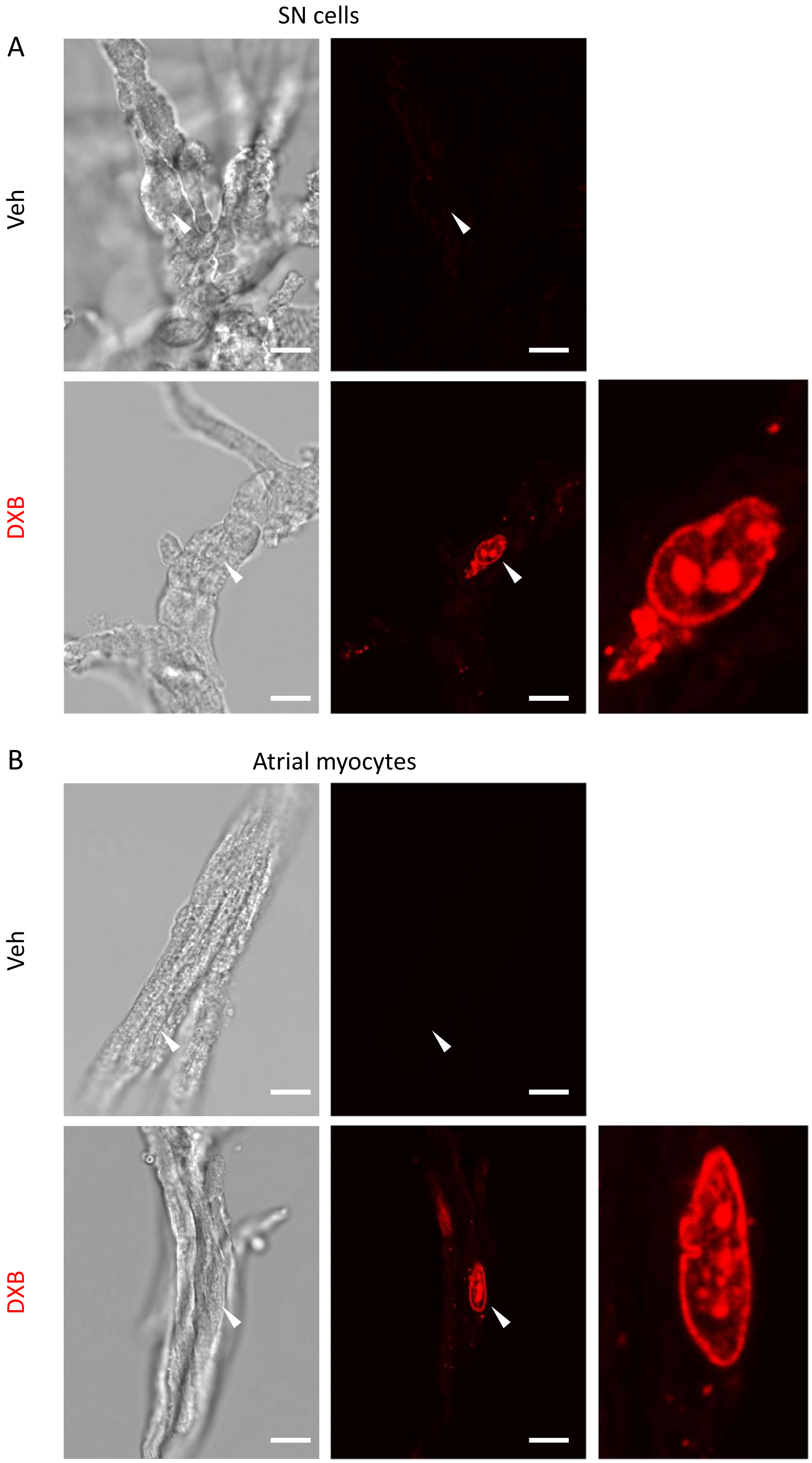

Fig. S4. Doxorubicin (DXB) accumulates the nuclei in sinus node (SN) and atrial myocytes.
A and B. Representative micrographs of sinus node (A) and atrial myocytes (B) isolated from vehicle (Veh)- and DXB-treated right atrial preparations. Arrowheads indicate the location of the nuclei. Scale bars = 10 μm. Enlarged images were from the perinuclear area of the neighbouring images.

Fig. S5

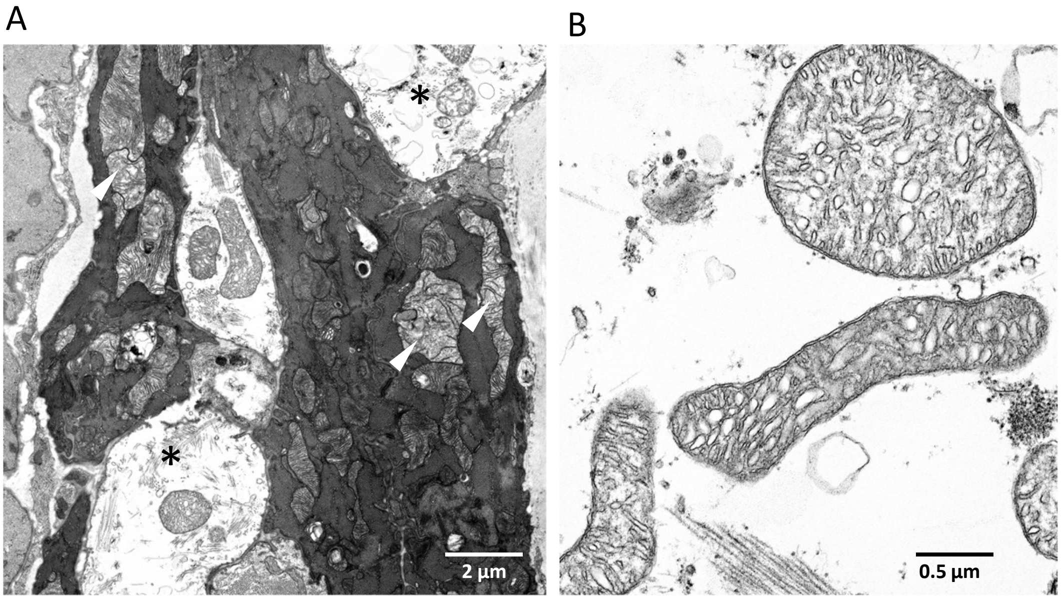

Fig. S5. A few numbers of sinus node cells are severely damaged by doxorubicin (DXB) treatment. **A.** Representative micrographs of impaired sinus node cells containing raptured myofibrils (*) and swollen mitochondria (arrowheads). **B.** An enlarged image of impaired mitochondria showing a low density of cristae.

Fig. S6

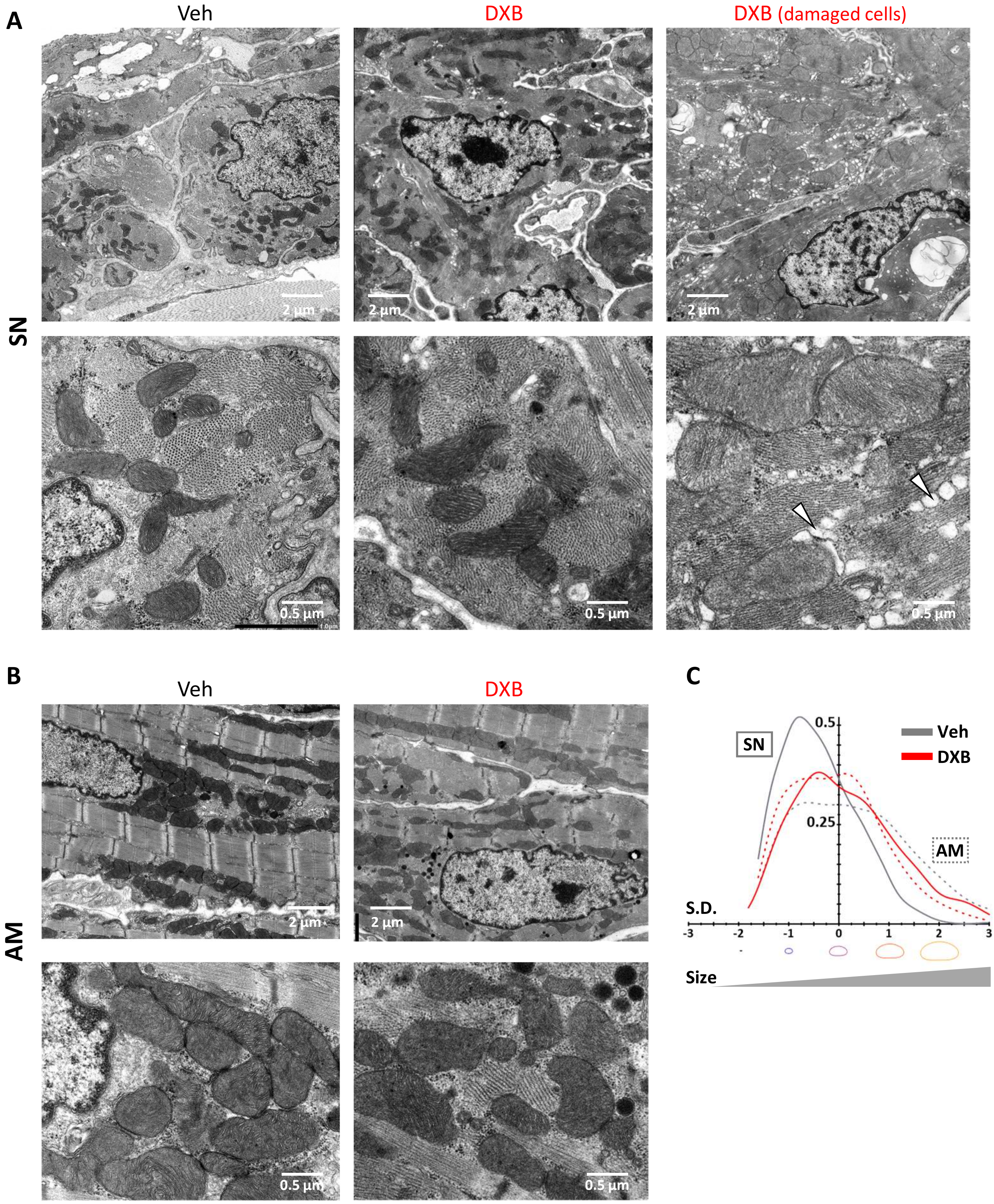

Fig. S6. Doxorubicin (DXB) causes mitochondrial impairment in sinus node cells (SN) and atrial myocytes (AM). A and B. Representative electronmicrographs of SN and AM in chronically vehicle (Veh)- or DXB-treated mice (n = 3/group). DXB samples includes severely damaged cells showing mitochondrial swelling and dilation of sarcoplasmic reticulum (arrow heads). **C.** Kernel density estimation of mitochondrial size distributions in SN and AM of Veh- or DXB-treated mice.
